# Ternary Full Adder Designs Employing Unary Operators and Ternary Multiplexers

**DOI:** 10.3390/mi14051064

**Published:** 2023-05-17

**Authors:** Ramzi A. Jaber, Ali M. Haidar, Abdallah Kassem, Furqan Zahoor

**Affiliations:** 1 Electrical and Electronic Engineering Department, Lebanese University, Hadath 40016, Lebanon; ramzi.jaber@ul.edu.lb; 2 Electrical and Computer Engineering Department, Beirut Arab University, Debieh 1504, Lebanon; ari@bau.edu.lb; 3 Electrical and Computer Engineering Department, Notre Dame University, Louaize 1201, Lebanon; akassem@ndu.edu.lb; 4 School of Computer Science and Engineering, Nanyang Technological University, Singapore 639798, Singapore

**Keywords:** CNFET, logic design, MVL, nanoscale devices, ternary full adder (TFA), ternary multiplexer (TMUX), unary operators

## Abstract

The design of the Ternary Full Adders (TFA) employing Carbon Nanotube Field-Effect Transistors (CNFET) has been widely presented in the literature. To obtain the optimal design of these ternary adders, we propose two new different designs, TFA1 with 59 CNFETs and TFA2 with 55 CNFETs, that use unary operator gates with two voltage supplies ($V_{dd}$ and $V_{dd}/2$) to reduce the transistor count and energy consumption. In addition, this paper proposes two 4-trit Ripple Carry Adders (RCA) based on the two proposed TFA1 and TFA2; we use the HSPICE simulator and 32 nm CNFET to simulate the proposed circuits under different voltages, temperatures, and output loads. The simulation results show the improvements of the designs in a reduction of over 41% in energy consumption (PDP), and over 64% in Energy Delay Product (EDP) compared to the best recent works in the literature.

## 1. Introduction

Due to the difficulties associated with the scaling of silicon transistors, various technologies have been investigated as the feasible alternatives. The existing complementary metal-oxide semiconductor (CMOS) technology faces many critical issues, such as high-power dissipation, short channel effects, and reduced gate control when scaled to nanoscale dimensions. These reliability issues significantly degrade the system’s performance. However, CNFETs seem to provide better performance because of their increased carrier velocity, excellent carrier mobility, and greater trans-conductance [1]. In addition, CNFETs offer great promise to the design of Multi-Valued Logic (MVL) circuits with the ability to adjust the desired threshold voltages.

In the last decade, many ternary circuit designs have been demonstrated using CNFET technology, such as ternary logic gates, memory, and combinational circuits [2,3,4,5,6]. More specifically, several ternary full adders have been proposed [7,8,9,10,11,12,13,14,15,16,17,18]. We compared the performance of these ternary adders with the proposed designs. The main objective of this work focuses on the design optimization of ternary adders. This paper uses CNFET transistors and an unbalanced ternary logic system (0 (0 v), 1 ($V_{dd}/2,2(V_{dd}$)) for implementing the designs.

### 1.1. How to Produce Logic 1 ($V_{dd}/2$) in Ternary Circuits?

The hard way is how to produce logic 1 in ternary circuits. One technique consists in using a voltage divider to generate logic 1 ($V_{dd}/2$) from one power supply ($V_{dd}$) by inserting two diode-connected transistors acting like resistors; however, this technique produces a direct current path from the power supply ($V_{dd}$) to the ground and generates static power dissipation as shown in Equation (1a) of the general equation of the total power consumption (1), whereas the dynamic power is shown in Equation (1b).

(1) $$P=P_{s}+P_{d}$$

(1a) $$Static:P_{s}=P_{leakage}+k1*N*V_{dd}^{2}/R$$

(1b) $$Dynamic:P_{d}=k2*\sum C_{i}*V_{dd}^{2}*f$$

where:

- *N*: Transistors count in the circuit,
- $V_{dd}$: Power Supply,
- $k_{1}$: Ratio of diode-connected transistors,
- *R*: Diode-connected transistor resistivity,
- $k_{2}$: Ratio of switching capacitors,
- $C_{i}$: Load Capacitor or Internal Capacitor,
- *f*: Clock frequency of the circuit.

**Figure 1:**
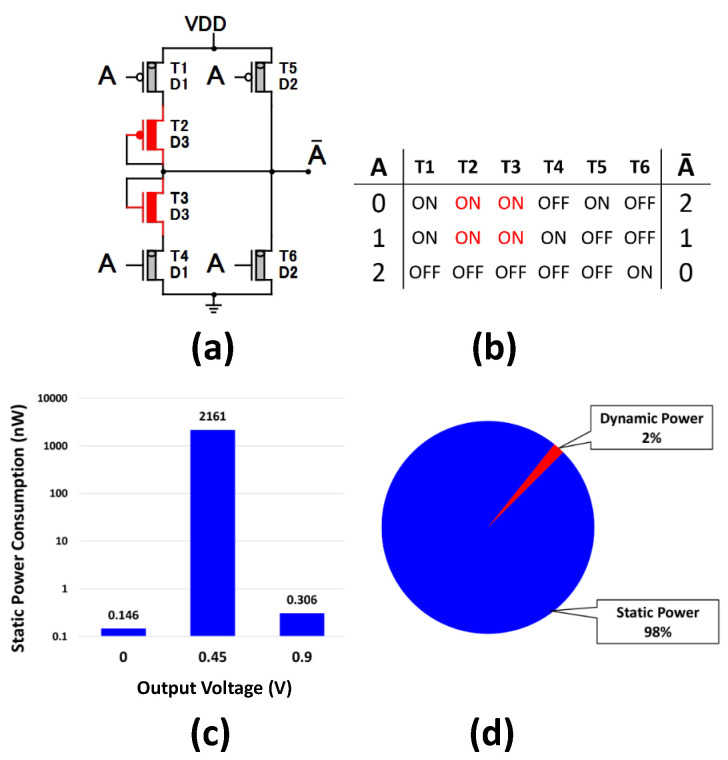
Measuring the static power of STI [7]: (**a**) STI Circuit, (**b**) STI truth table, and (**c**,**d**) showing that the static power is 98% of the average power consumption when logic 1 (0.45 V) is produced by two diode-connected transistors (T2, T3).

To illustrate that, in Figure 1, we will analyze the static power and the dynamic power of the Standard Ternary Inverter (STI) [7], which is a classic example of generating logic (1) from a single source.

In this paper, we use the alternative solution with two power supplies ($V_{dd}$ and $V_{dd}/2$) to remove these two diode-connected transistors to eliminate the static power; however, the drawback is the increase in interconnections.

### 1.2. Literature Review

Many articles proposed different methodologies to design TFAs based on CNFET. Table 1 presents the techniques and the limitations for the most important latest ones.

Additionally, we will describe them as follows:

(1) Implement the conventional design by converting the ternary inputs to intermediate binary bits using Ternary Decoders (TDecoders), then using binary gates, and, lastly, using ternary encoders to produce the final ternary outputs. This method will generate a high transistor count and PDP, as observed in the following papers:
  Authors of [7] created a TFA with 412 CNFETs. In [8], the authors presented a TFA with 337 CNFETs and 14 RRAMs (Resistive Random Access Memory).
(2) Use algorithms for logic synthesis. This strategy will result in a large transistors count connected in series, resulting in high propagation delays and PDP. Papers using this approach are:
  Authors of [9] represented a TFA with 105 CNFETs using two custom algorithms to generate unary operators and cascading TMUXs. Authors of [10] showed a TFA with 98 CNFETs using a Ternary-Transformed Binary Decision Diagram (TBDD) algorithm, and the authors of [11] represented a TFA with 106 CNFETs using a modified Quine–McCluskey and post-optimization algorithms.
(3) Use unary operators of the ternary system with TMUXs. It is the technique that we use in this paper. This method will generate a low transistor count and low PDP. The articles using this approach are:
  Authors of [12,13,14] designed TFAs with 74, 89, and 72 CNFETs, respectively.
(4) The following papers use mixed techniques:
  Authors of [15] proposed a TFA with 142 CNFETs using unary operators based on Binary NAND, TMUXs, and ternary encoders. In [17], the authors proposed a TFA with 74 CNFETs using PTL (Pass Transistor Logic) and TMUXs, which produce medium propagation delays and a medium PDP. The authors of [18] proposed a TFA with 54 CNFETs using unary operators, Transmission Gates, PTL, and TDecoders.

Finally, we will discuss the debatable approach. Authors of [16] represented two TFAs with 49 and 37 CNFETs using a capacitive network (the threshold logic approach). The drawback of this method is a drastic reduction in the noise margins when coherent noises are simultaneously present on the different inputs and high propagation delays and PDP. We will exclude these TFAs from the comparison with other TFAs. To our knowledge, a linear combination of inputs has not been used for binary logic circuits since the 1970s, when Resistor Transistor Logic (RTL) was replaced by Diode Transistor Logic (DTL). Replacing resistors with capacitors does not change the issue.

### 1.3. Contributions

The above designs have massive transistors count, high propagation delays, and (or) high PDP.

This paper proposes two TFAs with 59 and 55 CNFETs using unary ternary operators and TMUXs to obtain the lowest PDP.

Remark: Not always the reduction in the number of transistors is a good design. We must consider parameters such as (1) the critical path between the inputs and the outputs (see section “Design Methodology”); (2) the direct current path from the power supply to the ground, as described above. That is why we use unary ternary operators and TMUXs.

The following are the main contributions of this paper:

1. Not using the basic ternary logic gates (STI, TNAND, TNOR), TDecoders, and ternary encoders. Because using the basic ternary logic gates will produce a high transistor count and more energy consumption (compared to [7,8,15]).
2. Using unary operators can replace basic ternary logic gates, resulting in a considerable reduction in the number of transistors utilized and PDP.

## 2. CNFET Transistor

**Figure 2:**
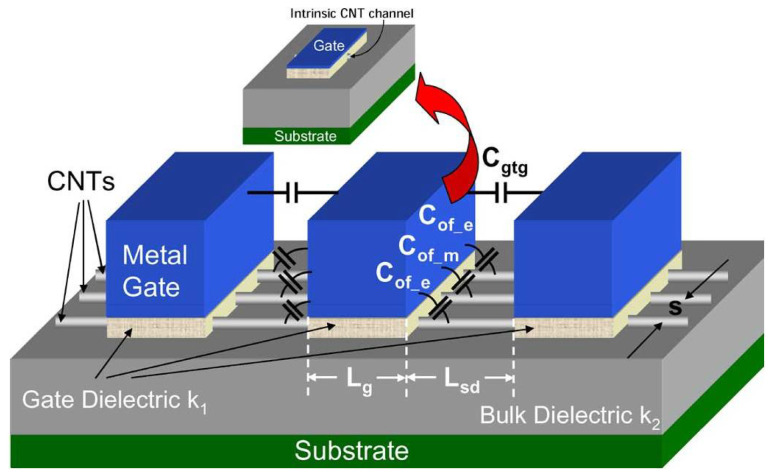
Stanford CNFET model [19]. The carbon nanotubes are below the gate.

This paper uses the Stanford CNFET model [19], as shown in Figure 2. However, the following Equation (2) shows that the threshold voltage depends on the diameter of the carbon nanotube (CNT):

(2) $$V_{\mathrm{th}}\approx \frac{0.43}{Dcnt}$$

where Dcnt is the CNT diameter.

Because we use an unbalanced ternary logic system (0 (0 v), 1 ($V_{dd}/2$), 2($V_{dd}$)) then we want to choose two threshold voltages to achieve three logic states from the CNFET. The best two threshold voltages are 0.289 V and 0.559 V, as described in Table 2.

Table 2 explains how the CNFET transistors work, as well as the relationship between the threshold voltage and the diameter of the carbon nanotubes that are used in this paper.

More information about CNFETs can be found in [19,20,21].

## 3. Design Methodology

This paper proposes two different TFAs using the proposed unary operators combined with two different TMUXs.

### 3.1. Two Proposed Unary Operators

The unary operators of a *m*-valued system are logic gates with one input and one output.

Table 3 shows seven unary functions to be used in the designs of TFAs. Where *A* is the ternary input, $A_{p}$ is a Positive Ternary Inverter (PTI), $A_{n}$ is a Negative Ternary Inverter (NTI), $A^{1}=\left(A+1\right)$ mod (3) called successor or single shift operator and $A^{2}=\left(A+2\right)$ mod (3) called Predecessor or dual shift operator are the cycle operators. $A_{1}$ is the decisive literal, and the last two unary functions are $1\cdot {\bar{A}}_{n}$ and $1\cdot {\bar{A}}_{p}$ [22].

**Figure 3:**
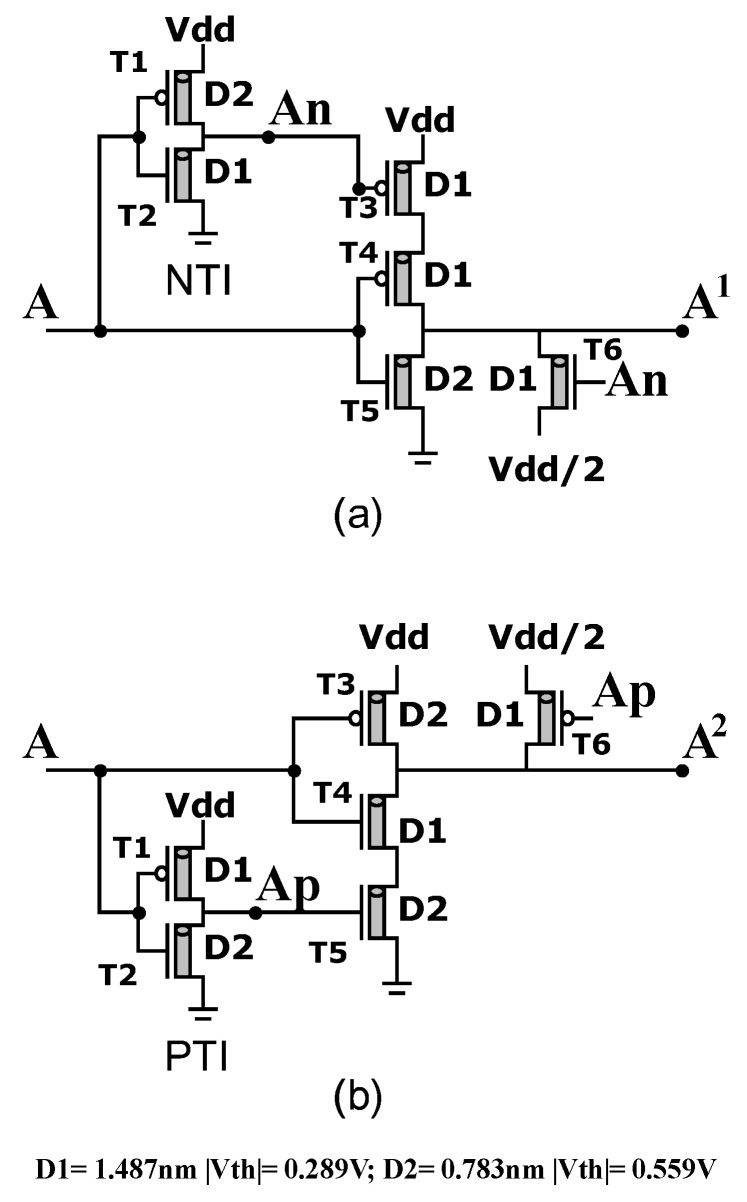
Proposed unary operators: (**a**) $A^{1}$ circuit: The input A enters NTI (T1, T2) and (T4, T5) then $A_{n}$ enters (T3, T6) to obtain output $A^{1}$. (**b**) $A^{2}$ circuit: The input A enters PTI (T1, T2) and (T3, T4) then $A_{p}$ enters (T5, T6) to obtain output $A^{2}$.

We propose new designs for two unary operators $A^{1}$ and $A^{2}$, as shown in Figure 3.

The other five unary operators are presented in [23,24].

The operations of the proposed unary operators are summarized in Table 4 and Table 5.

Table 6 shows the transistor count comparison of the proposed unary operators to those in [9,13,15,24].

### 3.2. Ternary Multiplexers

**Figure 4:**
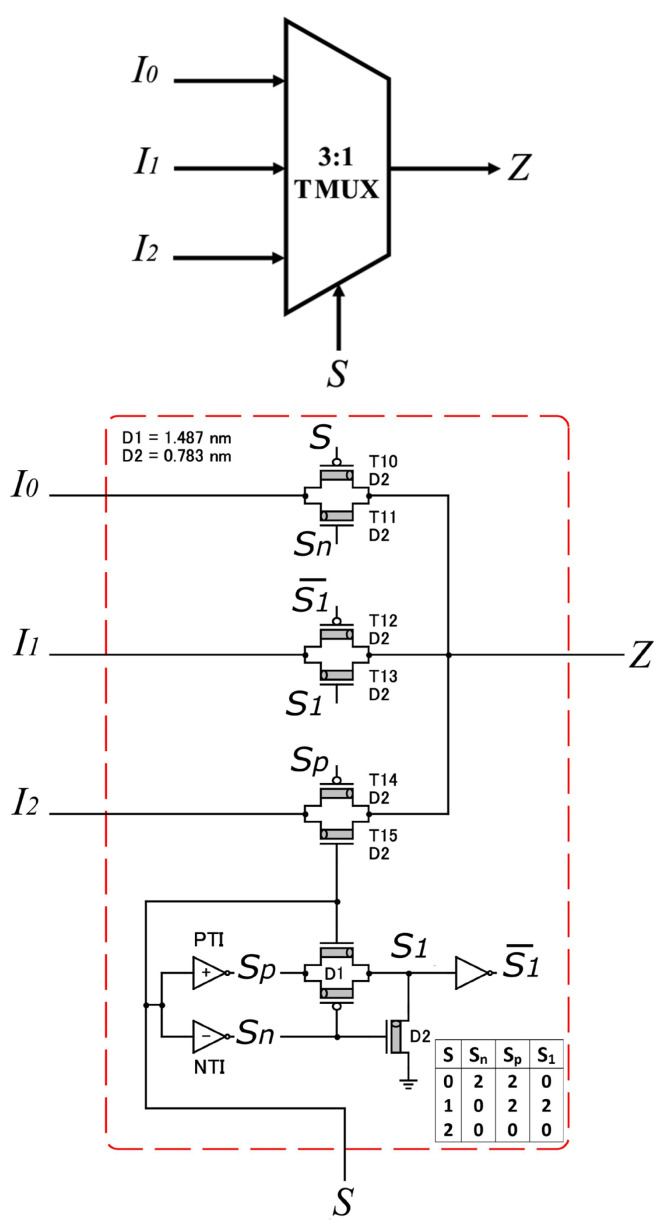
(3:1) TMUX in [23]. Three inputs enters the TMUX to produce one output as described in Equation (3).

Figure 4 shows the (3:1) TMUX [23] with 15 transistors. It has three inputs ($I_{0}$, $I_{1}$, $I_{2}$), one selection (*S*), and one output (*Z*), as described in Equation (3).

(3) $$Z=\left\{\begin{array}{cc} I_{0}, & if\;S=0\\ I_{1}, & if\;S=1\\ I_{2}, & if\;S=2 \end{array}\right.$$

**Figure 5:**
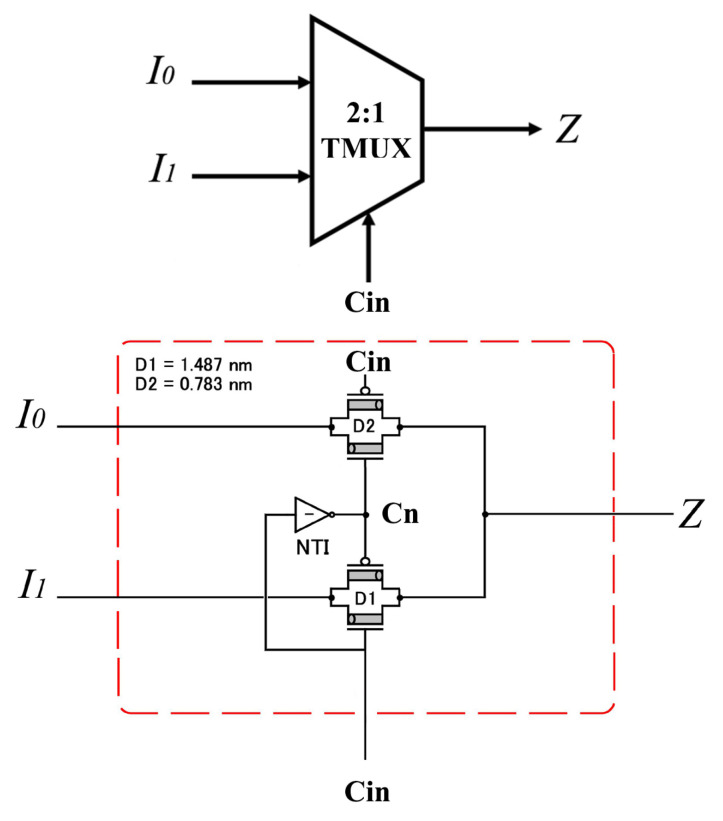
Special (2:1) TMUX for selection $C_{in}$. Two inputs enters the TMUX to produce one output as described in Equation (4).

The second TMUX has $C_{in}$ as a selection, which values are only 0 or 1 ($V_{dd}/2$). A special (2:1) TMUX with 6 transistors is presented in Figure 5, as described in Equation (4).

(4) $$Z=\left\{\begin{array}{cc} I_{0}, & if\;C_{in}=0\\ I_{1}, & if\;C_{in}=1 \end{array}\right.$$

Compared to the typical (2:1) Binary MUX, this special (2:1) Ternary MUX has a 0.289 V instead of 0.559 V threshold voltage for the second transmission gate. Cn is the NTI output of select input $C_{in}$ instead of $\bar{C}$ in (2:1) Binary MUX.

### 3.3. Proposed Two TFAs

A 1-trit Ternary Full Adder adds three ternary inputs (*A*, *B*, and $C_{in}$ (Carry-in)) and produces two outputs, the Sum and the Carry Out ($C_{out}$), as described in Table 7. $C_{in}$ has only values 0 or 1 ($V_{dd}/2$).

The general equations of the Sum and the Carry Out ($C_{out}$) are shown in (5):

(5) $$\begin{array}{c} Sum=\left(A+B+C_{in}\right)\;mod\left(3\right)\\ C_{out}=\left\lfloor \left(A+B+C_{in}\right)/3\right\rfloor \end{array}$$

Equations (6) and (7) are derived from Table 7. Using unary operators and TMUXs, they are:

(6) $$Sum=\left\{\begin{array}{cc} A\cdot B_{0}+A^{1}\cdot B_{1}+A^{2}\cdot B_{2} & if\;C_{in}=0\\ A^{1}\cdot B_{0}+A^{2}\cdot B_{1}+A\cdot B_{2} & if\;C_{in}=1 \end{array}\right.$$

(7) $$\begin{array}{c} C_{out}=\left\{\begin{array}{cc} 0\cdot B_{0}+\left(1\cdot {\bar{A}}_{p}\right)\cdot B_{1}+\left(1\cdot {\bar{A}}_{n}\right)\cdot B_{2} & if\;C_{in}=0\\ \left(1\cdot {\bar{A}}_{p}\right)\cdot B_{0}+\left(1\cdot {\bar{A}}_{n}\right)\cdot B_{1}+1\cdot B_{2} & if\;C_{in}=1 \end{array}\right. \end{array}$$

where

(8) $$B_{i}=\left\{\begin{array}{cc} 2 & if\;B=i\\ 0 & if\;B\neq i \end{array}\right.$$

#### 3.3.1. First Proposed TFA1

We use in this design the following technique: starting with unary operators, (2:1) TMUXs and (3:1) TMUXs.

**Figure 6:**
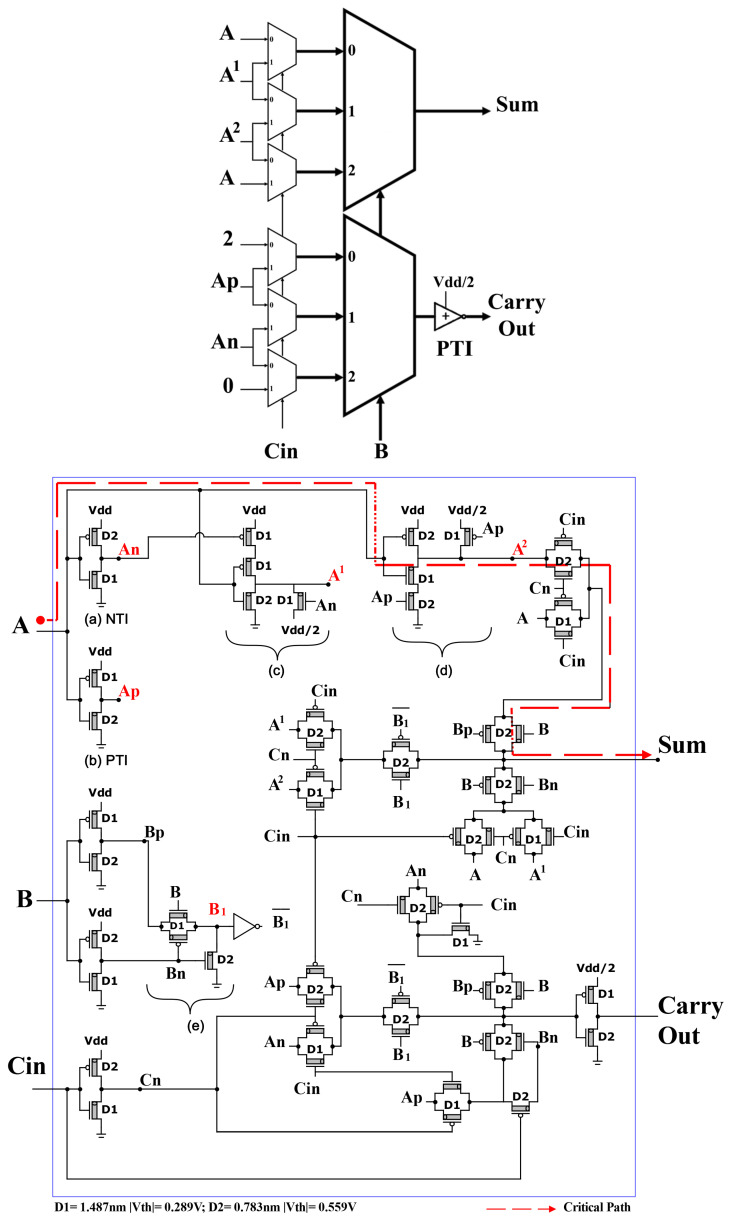
Proposed TFA1 with 59 CNFETs. Unary operators sub-circuits are: (**a**) NTI, (**b**) PTI, (**c**) $A^{1}$, (**d**) $A^{2}$, and (**e**) $B_{1}$.

The three ternary inputs (*A*, *B*, $C_{in}$) enter the six unary operators sub-circuits. Then the outputs of unary operators enter the special (2:1) TMUXs, and the outputs of (2:1) TMUXs enter the (3:1) TMUXs to produce the final outputs (Sum and Carry Out), as shown in Figure 6. The critical path (dotted red line) is the maximum propagation delay from the input “*A*” to the output “Sum” via ($A,Ap,A^{2}$, first TG in (2:1) TMUX, third TG in (3:1) TMUX, then Sum) when “*A*” changes from 1 to 2, “*B*” = 2, “$C_{in}$” = 0, and “Sum” from 0 to 1. The path from $C_{in}$ to $C_{out}$ is the critical one in N-trit carry propagate adders (see Section 3.3.2).

#### 3.3.2. Second Proposed TFA2

We use the other technique in this design, starting with unary operators, (3:1) TMUXs, and (2:1) TMUXs.

**Figure 7:**
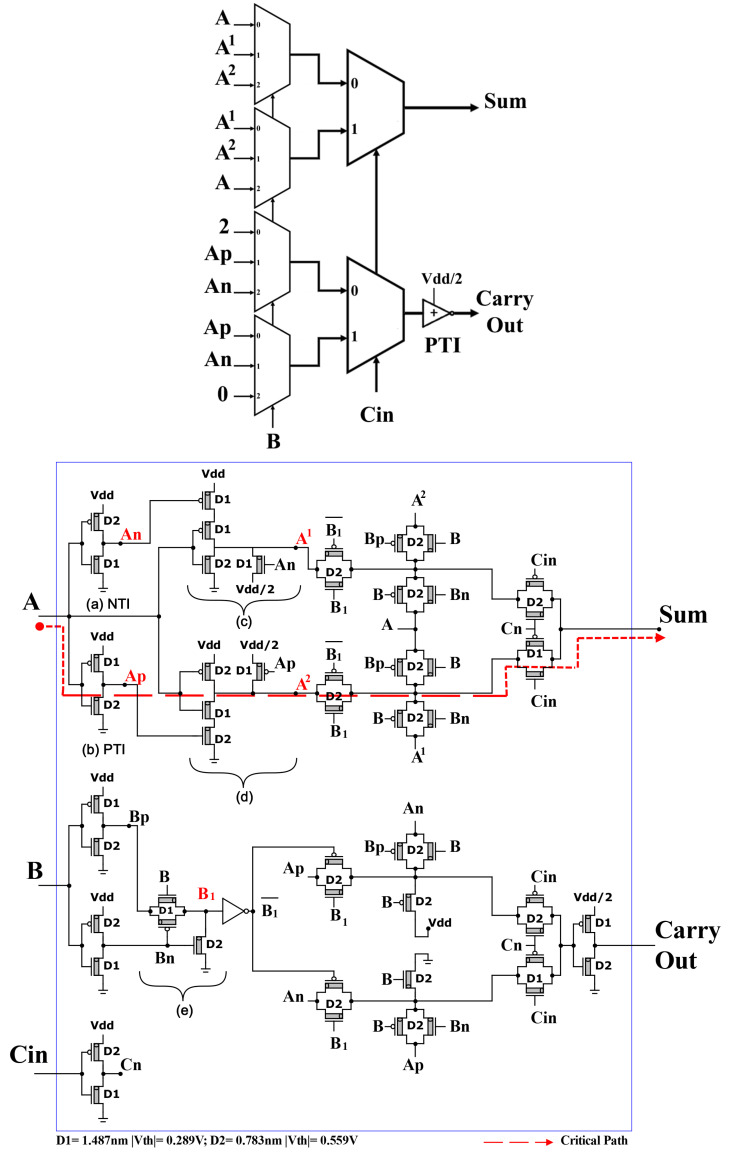
Proposed TFA2 with 55 CNFETs. Unary operators sub-circuits are: (**a**) NTI, (**b**) PTI, (**c**) $A^{1}$, (**d**) $A^{2}$, and (**e**) $B_{1}$.

The three ternary inputs (*A*, *B*, $C_{in}$) enter the unary operators sub-circuits. Then the outputs of unary operators enter the (3:1) TMUXs, and the outputs of (3:1) TMUXs enter the special (2:1) TMUXs to produce the final outputs (Sum and Carry Out), as shown in Figure 7.

The critical path (dotted red line) from the input *A* to the output Sum via ($A,Ap,A^{2}$, second TG in (3:1) TMUX, second TG in (2:1) TMUX, then Sum) when “*A*” changes from 1 to 2, “*B* = 1”, “$C_{in}$” = 1, and “Sum” from 0 to 1.

The propagation delay in the critical path of TFA2 is less than the one of TFA1, as observed by comparing Figure 6 and Figure 7.

#### 3.3.3. 4-Trit Ripple Carry Adder

A Ripple Carry Adder (RCA) is a logic circuit that cascades multiple full adder circuits. The carry-out of each full adder is the carry-in of the next one.

**Figure 8:**
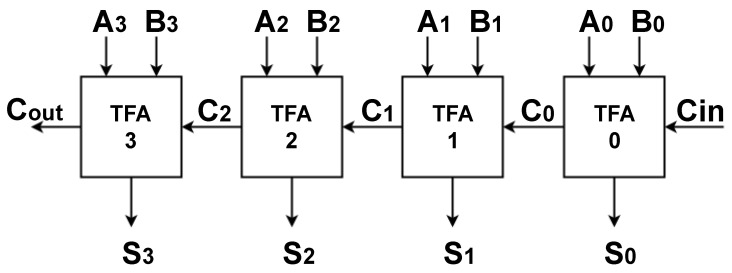
4-Trit Ripple Carry Adder Model that cascades 4 TFAs. The critical path is from $C_{in}$ to $C_{out}$.

This paper proposed two 4-trit RCAs using the proposed TFAs to demonstrate the efficiency of the proposed circuits in the design of larger computational blocks. The general model of the proposed 4-trit RCA is shown in Figure 8. The critical path in N-trit RCA is always from $C_{in}$ to $C_{out}$.

## 4. Results and Discussion

The proposed TFAs are simulated and compared to 32 nm channel CNFET-Based TFAs in [7,8,9,10,11,12,13,15,16,17,18] using the HSPICE simulator.

**Figure 9:**
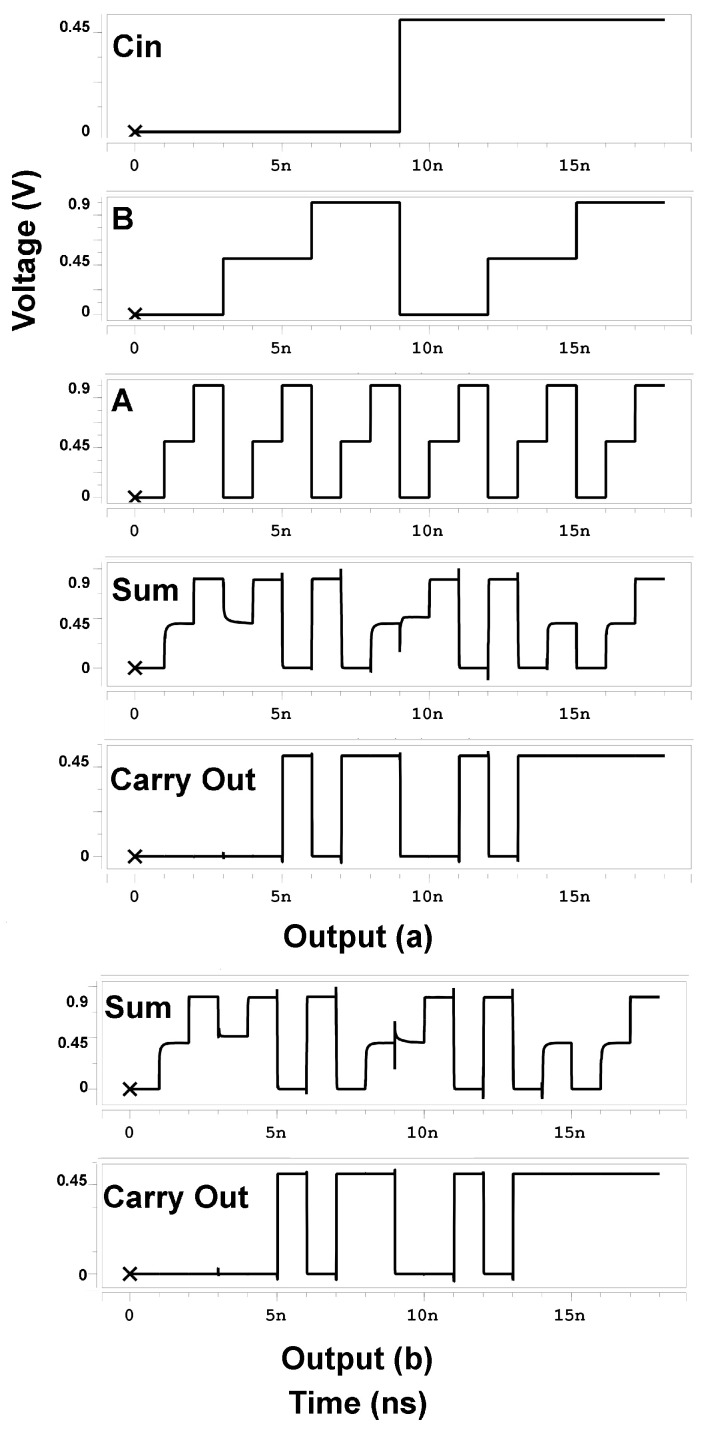
Wave form of the proposed: (**a**) TFA1 and (**b**) TFA2. Two inputs and a Carry-in ($A,B,C_{in}$) with all their different values studied. To produce two outputs Sum and the Carry out.

The simulation parameters for Figure 9 and Table 8 are $V_{dd}$ = 0.9 V, temperature = 27 °C, frequency = 1 GHz, and fall/rise time = 20 ps for all input signals.

Figure 9 shows the transient analysis of the proposed (a) TFA1 and (b) TFA2.

Table 8 compares all the investigated TFA circuits regarding transistor count, average power, maximum delay, maximum PDP (Power Delay Product), and maximum EDP (Energy Delay Product). The values in bold are the lowest values (best values). Because the results of the proposed TFA2 are better than the proposed TFA1, we will compare the proposed TFA2 to the other designs. The comparison to the lowest value (bolded or *) inside each column regarding the proposed TFA2 using the comparison ratio value Equation (9).

(9) Ratio=Bestpreviousdesign/Proposeddesign

where Ratio>1: It means that the proposed design is better.

The results show that the proposed TFA2 is better than the best other designs regarding max. propagation Delays, PDP, and EDP.

### 4.1. Different Voltages, Temperatures, and Output Loads

To study the performance and efficiency of the proposed circuits, we simulate the proposed TFA1 and TFA2 for different voltages (Table 9), different temperatures (Table 10), and different output loads (Table 11).

**Figure 10:**
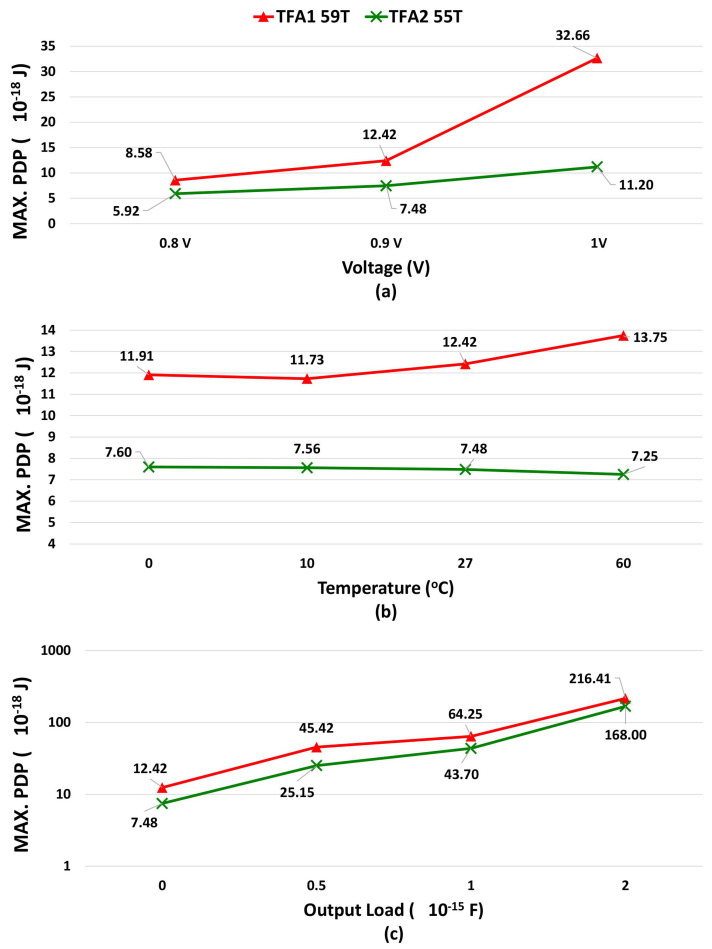
MAX. PDP Comparison: (**a**) Voltage Variations, (**b**) Temperature Variations, and (**c**) Output Load Variations: showing the MAX. PDP comparision between the proposed TFAs for different voltage, temperature, and output load.

**Figure 11:**
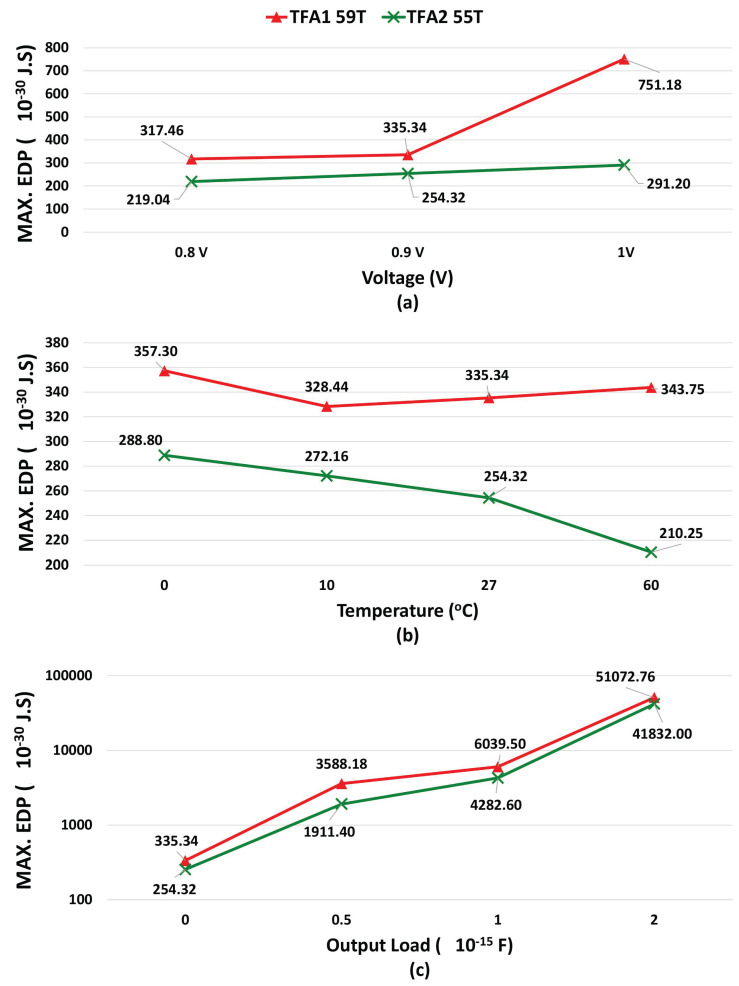
MAX. EDP Comparison: (**a**) Voltage Variations, (**b**) Temperature Variations, and (**c**) Output Load Variations: showing the MAX. EDP comparison between the proposed TFAs for different voltage, temperature, and output load.

In addition, we simulate the proposed TFAs regarding maximum PDP, and maximum EDP, as shown in Figure 10 and Figure 11, (a) voltage variations, (b) temperature variations, and (c) output load variations.

As shown in Table 9, Table 10 and Table 11 and in Figure 10 and Figure 11, the proposed TFA2 gives lower results compared to TFA1 in all study parameters, lower power, lower propagation delay (more speed), and lower energy consumption. Therefore, the proposed TFA2 is more stable and better than the proposed TFA1.

We prove that to design TFA using (3:1) TMUX then (2:1) TMUX will give better performance than the design using (2:1) TMUX then (3:1) TMUX.

### 4.2. Scalability Study

We implement a 4-trit Ripple Carry Adder for each TFA design and simulate them with temperature at 27 °C, power supply at 0.9 V, frequency at 1 GHz, and fall and rise time of 20 ps, as shown in Table 12.

As shown in Table 12, the proposed 4-trit RCA that uses TFA2 has better performance than others. Therefore, the proposed TFA2 can be used for larger adders.

## 5. Conclusions

This paper proposes two novel 32 nm channel CNFET-based designs of Unary Operators combined with a Ternary Multiplexer to design two different Ternary Full Adders.

The design process uses different techniques regarding transistor arrangement, two voltage supplies ($V_{dd}$, $V_{dd}/2$), and a transistor count reduction to lower the energy consumption of the ternary full adder.

Compared to recent similar designs, the HSPICE simulation results show higher performance and lower energy consumption.

It seems that these designs are closed to the optimal design of ternary adders. This work will be continued by the design of quaternary adders and multipliers to examine how the performance evolves when moving from ternary to quaternary circuits. These ternary and quaternary arithmetic circuits will be compared with the corresponding binary ones.

## Figures and Tables

**Table 1:** Literature review summary: presenting the techniques and the limitations for the most important designs.

Techniques	Refs.	Year	Details	CNFET # TFA	Limitation
Conventional Design	[7]	2011	- TDecoders (16 transistors)	412	
			- Binary gates		
			- Ternary encoder		- High transistor count
	[8]	2021	- TDecoders (10 transistors)	337	- High PDP
			- Binary gates		
			- 14 RRAMs		
Algorithms Synthesis	[9]	2017	- Two custom Algorithms	105	
			- Cascading TMUXs		- Produce a large number of transistors in series
	[10]	2018	- TBDD Algorithm	98	- High Propagation Delays
	[11]	2020	- Modified Quine-McCluskey Algorithm	106	- High PDP
Unary Operators and TMUXs	[12]	2017	- TMUXs (12 transistors)	74	- Cascading Transmission Gates
			- Two voltage supplies ($V_{dd}$, $V_{dd}/2$)		- High Propagation Delays and PDP
	[13]	2018	- TMUXs (22 transistors)	89	- High transistor count
			- Two voltage supplies ($V_{dd}$, $V_{dd}/2$)		
	[14]	2021	- TMUXs (15 transistors)	72	
Other or Mixed Designs	[15]	2019	- Unary Operators based on Binary NAND	142	
			- TMUXs (18 transistors)		- High transistor count and PDP
			- Ternary encoders		
	[16]	2020	- Two designs	49	- Drastic reduction in the noise margins
			- Capacitive network	37	- High Propagation Delays
			- STI inverter		- High PDP
	[17]	2021	- Pass Transistor Logics	74	
			- TMUXs (12 transistors)		
	[18]	2021	- Unary Operators	54	- Medium Propagation Delays
			- TDecoders		- Medium PDP
			- Transmission Gates		
			- Pass Transistor Logics		

**Table 2:** Operation of CNFET with D1 = 1.487 nm and D2 = 0.783 nm. Showing when the transistor will open and close.

		Threshold	Voltage Gate		
Type	Diameter	Voltage	0 V	0.45 V	0.9 V
P-CNFET	D1	−0.289 V	ON	ON	OFF
	D2	−0.559 V	ON	OFF	OFF
N-CNFET	D1	0.289 V	OFF	ON	ON
	D2	0.559 V	OFF	OFF	ON

**Table 3:** Truth table of the selected Unary Operators: $A_{p}$, $A_{n}$, $A^{1}$, $A^{2}$, $A_{1}$, $1\cdot {\bar{A}}_{n}$, and $1\cdot {\bar{A}}_{p}$.

Ternary	PTI	NTI	Cycle Operators		Decisive		
Input *A*	$A_{p}$	$A_{n}$	$A^{1}$	$A^{2}$	Literal $A_{1}$	$1\cdot {\bar{A}}_{n}$	$1\cdot {\bar{A}}_{p}$
0	2	2	1	2	0	0	0
1	2	0	2	0	2	1	0
2	0	0	0	1	0	1	1

**Table 4:** Truth Table and operation of the circuit $A^{1}$.

	Transistors							Output
*A*	T1	T2	$A_{n}$	T3	T4	T5	T6	$A^{1}=\left(A+1\right)$ Mod (3)
0	ON	OFF	2	OFF	ON	OFF	ON	1
1	OFF	ON	0	ON	ON	OFF	OFF	2
2	OFF	ON	0	ON	OFF	ON	OFF	0

**Table 5:** Truth Table and operation of the circuit $A^{2}$.

	Transistors							Output
*A*	T1	T2	$A_{p}$	T3	T4	T5	T6	$A^{2}=\left(A+2\right)$ Mod (3)
0	ON	OFF	2	ON	OFF	ON	OFF	2
1	ON	OFF	2	OFF	ON	ON	OFF	0
2	OFF	ON	0	OFF	ON	OFF	ON	1

**Table 6:** Unary Operators transistor count comparison. Showing the transistor count comparison of the proposed unary operators among others.

	[9]	[13]	[15]	[24]	Proposed
$A^{1}$	7	17	10	6	6
$A^{2}$	7	17	10	11	6
**Total**	14	34	20	17	12
Improvement	14%	65%	40%	29%	-

**Table 7:** 1-trit TFA truth table.

$C_{\mathrm{in}}$	*B*	*A*	Sum	Carry Out
**0**	**0**	$\begin{array}{c} 0\\ 1\\ 2 \end{array}$	$\begin{array}{c} 0\\ 1\\ 2 \end{array}\}A$	$\begin{array}{c} 0\\ 0\\ 0 \end{array}\}0$
	**1**	$\begin{array}{c} 0\\ 1\\ 2 \end{array}$	$\begin{array}{c} 1\\ 2\\ 0 \end{array}\}A^{1}$	$\begin{array}{c} 0\\ 0\\ 1 \end{array}\}1\cdot {\bar{A}}_{p}$
	**2**	$\begin{array}{c} 0\\ 1\\ 2 \end{array}$	$\begin{array}{c} 2\\ 0\\ 1 \end{array}\}A^{2}$	$\begin{array}{c} 0\\ 1\\ 1 \end{array}\}1\cdot {\bar{A}}_{n}$
**1**	**0**	$\begin{array}{c} 0\\ 1\\ 2 \end{array}$	$\begin{array}{c} 1\\ 2\\ 0 \end{array}\}A^{1}$	$\begin{array}{c} 0\\ 0\\ 1 \end{array}\}1\cdot {\bar{A}}_{p}$
	**1**	$\begin{array}{c} 0\\ 1\\ 2 \end{array}$	$\begin{array}{c} 2\\ 0\\ 1 \end{array}\}A^{2}$	$\begin{array}{c} 0\\ 1\\ 1 \end{array}\}1\cdot {\bar{A}}_{n}$
	**2**	$\begin{array}{c} 0\\ 1\\ 2 \end{array}$	$\begin{array}{c} 0\\ 1\\ 2 \end{array}\}A$	$\begin{array}{c} 1\\ 1\\ 1 \end{array}\}1$

**Table 8:** TFAs Comparison: showing all the investigated TFA circuits with the proposed TFAs regarding transistor count, average power, maximum delay, PDP, and EDP.

	CNFETs	Power	Max.	Max. PDP	Max. EDP
TFA/Year	Count	(μW)	Delay (ps)	(×$10^{-18}$ J)	(×$10^{-27}$ J·s)
In [7] 2011	412	1.36	88	120	10.5
In [8] 2021	337	1.96	78	153	11.9
In [10] 2018	98	0.16	192	31	5.9
In [11] 2020	106	**0.13**	269	35	9.4
In [9] 2017	105	1.13	68	77	5.2
In [12] 2017	74	0.82	146	120	17.5
In [13] 2018	89	0.44	48	21	1
In [14] 2021	72	0.28	51	14.3	0.7 *
In [15] 2019	142	4.62	94	434	40.8
In [16] 2020	49	1.23	192	236	45.3
In [16] Design 2	**37**	0.81	262	212	55.5
In [17] 2021	74	**0.13**	98	12.75 *	1.2
In [18] 2021	54	0.43	47 *	20	0.9
**Proposed TFA1**	59	0.46	**27**	12.42	0.3
**Proposed TFA2**	55	0.22	34	**7.48**	**0.25**
Comparison to the lowest value (bolded or *) inside each column w.r.t. proposed TFA2					
Ratio = (Best previous value/proposed value); TFA2 is better for ratio > 1					
Comparison Ratio	0.67	0.59	1.38	1.70	2.8

**Table 9:** Voltage variations: showing the proposed TFAs for different voltages regarding average power, delay, PDP, and EDP.

TFA1 59T	Avg. Power	Avg.	Avg. PDP	Avg. EDP
$V_{\mathrm{dd}}$	(μW)	Delay (ps)	(×$10^{-18}$ J)	(×$10^{-30}$ J·s)
0.8 V	0.23	27.0	6.29	170
0.9 V	0.46	13.8	6.37	87.9
1 V	1.42	11.9	17	202
TFA2 55T				
0.8 V	0.16	32.8	5.25	172.2
0.9 V	0.22	14.0	3.10	43.4
1 V	0.43	12.0	5.16	61.9

**Table 10:** Temperature variations: showing the proposed TFAs for different temperatures regarding average power, delay, PDP, and EDP.

TFA1 59T	Avg. Power	Avg.	Avg. PDP	Avg. EDP
Temp.	(μW)	Delay (ps)	(×$10^{-18}$ J)	(×$10^{-30}$ J·s)
0°C	0.39	14.8	5.77	85.4
10°C	0.42	14.4	6.00	86.4
27°C	0.46	13.8	6.37	87.9
60°C	0.55	12.8	7.12	91.1
TFA2 55T				
0°C	0.20	15.2	3.05	46.3
10°C	0.21	14.7	3.09	45.4
27°C	0.22	14.0	3.10	43.4
60°C	0.25	12.9	3.20	41.3

**Table 11:** Output load variations: showing the proposed TFAs for different output load regarding average power, delay, PDP, and EDP.

TFA1 59T	Avg. Power	Avg.	Avg. PDP	Avg. EDP
Load (×$10^{-15}$ F)	(μW)	Delay (ps)	(×$10^{-18}$ J)	(×$10^{-27}$ J·s)
0 fF	0.46	13.8	6.37	0.09
0.5 fF	0.57	43.7	24.9	1.09
1 fF	0.68	71.8	48.8	3.50
2 fF	0.92	128	117.7	15.06
TFA2 55T				
0 fF	0.22	14.0	3.10	0.04
0.5 fF	0.33	45.5	15.0	0.69
1 fF	0.44	75.0	33.0	2.48
2 fF	0.67	134	89.8	12.0

**Table 12:** 4-Trit Ripple Carry Adders Comparison: comparing all the investigated RCA circuits with the proposed RCAs regarding transistor count, average power, maximum delay, PDP, and EDP.

		Avg.	Maximum		
	CNFET	Power	Delay	PDP	EDP
4-Trit RCA	Count	(μW)	(ps)	(×$10^{-18}$ J)	(×$10^{-27}$ J·s)
In [10] 2018	384	0.72	400	288	115
In [17] 2021	296	**0.55**	290	160	46
In [18] 2021	**216**	1.75	132	231	30
Proposed 1	236	2	135	270	36
Proposed 2	220	0.92	**84**	**77**	**6**
Previous TFA/proposed TFA2 Ratio					
TFA2 is better when ratio > 1					
w.r.t [10]	1.75	0.78	3.75	3.75	19
w.r.t [17]	1.35	0.60	3.45	2.08	7.67
w.r.t [18]	0.98	1.90	1.57	3.51	5

## Data Availability

Not applicable.

## Abbreviations

The following abbreviations are used in this manuscript:

CMOS	Complementary Metal-Oxide Semiconductor
FinFet	Field-Effect Transistor
CNFET	Carbon Nanotube Field-Effect Transistor
TFA	Ternary Full Adder
RCA	Ripple Carry Adder
PDP	Power Delay Product
EDP	Energy Delay Product
TMUX	Ternary Multiplexer
MVL	Multiple Valued Logic
RRAM	Resistive Random Access Memory
STI	Standard Ternary Inverter
PTI	Positive Ternary Inverter
NTI	Negative Ternary Inverter
TDecoder	Ternary Decoder
TBDD	Ternary-Transformed Binary Decision Diagram
PTL	Pass Transistor Logic
TG	Transmission Gate
RTL	Resistor Transistor Logic
DTL	Diode Transistor Logic
TNAND	Ternary AND
TNOR	Ternary OR
